# Recent advances in understanding and managing chordomas: an update

**DOI:** 10.12688/f1000research.22440.1

**Published:** 2020-07-16

**Authors:** Scott W. Connors, Salah G. Aoun, Chen Shi, Valery Peinado-Reyes, Kristen Hall, Carlos A. Bagley

**Affiliations:** 1 Department of Neurological Surgery, University of Texas Southwestern School of Medicine, Dallas, TX, USA; 2 UT Southwestern Spine Center, University of Texas Southwestern School of Medicine, Dallas, TX, USA; 3 Department of Orthopedic Surgery, University of Texas Southwestern School of Medicine, Dallas, TX, USA

**Keywords:** chordoma, primary bone tumors, targeted therapy, chordoma genetics, radiation therapy, chordoma surgery

## Abstract

Chordomas are rare and difficult-to-treat tumors arising from the embryonic notochord. While surgery is the mainstay of treatment, and despite new techniques aimed at maximizing total tumoral resection, recurrence remains high and the probability of disease-free survival low. New breakthroughs in genetics, targeted molecular therapy, and heavy-particle beam therapy offer some promise as adjuvant treatments in addition to surgical resection. A multidisciplinary approach encompassing genetics, immunotherapy, radiation therapy, and surgery, at a facility experienced in the management of this complex disease, offers the best chance of survival and quality of life to patients while limiting the intrinsic morbidity of these treatments.

## Introduction

Chordomas represent a complex clinical entity for which a definitive cure continues to elude clinicians despite a comprehensive multidisciplinary approach and a histologically benign pathology. Although they were described more than 150 years ago by Rudolf Virchow, who noted a slimy growth on the dorsum sellae during an autopsy ^1^, progress in understanding this disease has suffered, largely owing to its rarity. Chordomas have an incidence of only eight cases per million people per year and represent 1–4% of primary bone malignancies ^2^. Their locally destructive nature and their metastatic potential can lead to devastating outcomes with a median survival span of 10 years after diagnosis, although prognosis seems to be more favorable for spinal disease ^2, 3^. Despite these challenges, recent advances in molecular biology, genetics, and stereotactic beam therapy have the potential to improve our understanding of the pathogenesis of this disease and to provide viable targets for disease control. **

## Clinical features

Chordomas arise from notochord remnants and are found in the midline axis spanning from the apex of the skull base at the dorsum sellae to the anchor of the spine at the sacrum ^4^. While historically the mobile spine was considered only a rare site of chordoma formation, the Surveillance, Epidemiology, and End Results (SEER) database suggests a relative parity among the skull base (32%), mobile spine (32.8%), and sacrum (29%) ^2^.

Epidemiological studies show a 3:2 male predominance, with a median age at diagnosis of the mid to late fifth decade, although the range can be expansive, with recorded ages between 3 and 95 years old ^2, 5^. There also appears to be racial disparity, with Caucasians and Hispanics being more commonly affected than African Americans.

Because of chordomas’ slow growth, their signs and symptoms are most often the result of mass effect or local invasion, as the tumor can insidiously grow undetected for extended periods of time. Intracranial chordomas are often discovered during a workup for headache, neck pain, or neurological deficits such as double vision ^6, 7^. On the other hand, spinal lesions are more often associated with local pain or bowel and urinary dysfunction because of slow compression by space-occupying masses but can also result in neurological impingement with myelopathy or radiculopathy.

Prompt diagnosis is often difficult, as the initial symptoms are often insidious in their onset and lead to an array of gastrointestinal, urinary, or vague neurological complaints. This may delay patients seeking care, as well as postpone the prescription of diagnostic medical imaging. Radiographic evaluation is essential for lesion diagnosis, for assessing the degree of local tissue invasion and destruction, and, more importantly, for surgical planning.

Chordomas are erosive lesions that may display surrounding areas of soft tissue calcification on plain X-rays, as well as local destructive bony patterns, although simple X-ray films are of historical interest in the workup process today ^8^. On computed tomography, these lesions are well-demarcated soft tissue masses that demonstrate extensive osteolysis ^9^. The presence of bony destruction and extra-osseous extension can help to differentiate chordomas from other entities such as osteosarcomas and osteochondromas ^10^. Magnetic resonance imaging remains the mainstay of diagnostic evaluation for these tumors. Chordomas show intermediate to low signal intensity on T1 sequences with high intensity on T2 signal and appear very bright (T2 hyperintensity), likely because of the high fluid content of its vacuolated cellular components ^10^. Heterogeneous contrast enhancement is typical, with what is often described as a honeycomb appearance ^9, 10^, although the tumor may be non-enhancing. Obtaining a sample for pathological analysis is key for a final diagnosis and should be done prior to the initiation of treatment efforts, usually through an image-guided core biopsy. Proper biopsy techniques have to be respected, and the biopsy should be performed at centers with experience in performing them and with the capability of marking the biopsy tract for en bloc resection.

Location also has significant implications in the diagnosis and treatment of chordoma-like lesions on imaging. While it is often easy to obtain a percutaneous biopsy of spine lesions, this process can be challenging when addressing cranial masses given their proximity to vital nerves and structures. A lack of diagnosis via biopsy before proceeding with a surgical or radiosurgical solution can be problematic, as the differential diagnosis of cranial masses includes chondrosarcoma and ecchordosis physaliphora, which both have different treatment algorithms ^11^.

## Pathogenesis and genetics

Grossly, chordomas tend to appear as a lobulated mass with a gelatinous texture. Microscopically, these masses comprise distinct chords of cells contained in a myxoid matrix. The pathognomonic cellular features are physaliferous cells (or “bubble” cells) which contain intracytoplasmic vacuoles ^6^.

The relationship between the notochord and chordomas was initially suggested by German anatomist Johannes Peter Muller in 1858. He based his presumption on the fact that chordomas tended to be discovered in areas where notochord remnants were known to reside. Despite his highly prescient assertion, his theory was dismissed by his colleagues for want of evidence ^1^. Subsequent investigation, however, has given credence to Muller, supporting the notochordal origin of chordomas. The strongest evidence for this connection is genetic and relates to the Brachyury protein oncogene. This oncogene is located on chromosome 6q27 and is a T-box transcription factor involved in the differentiation of notochordal tissue ^12, 13^. Vujovic *et al*. found Brachyury expressed in all 53 cases of chordomas in their series and described it as a biomarker for the disease ^14^. Brachyury has also been identified as a susceptibility gene in families with hereditary chordoma ^4, 15^, and short hairpin ribonucleic acid (shRNA) silencing of its oncogene has led to *in vitro* growth arrest in patient-derived cell lines ^12, 15^. Brachyury expression may also have prognostic implications, as the duration of progression-free survival may be shorter in patients with higher levels of Brachyury protein expression ^12^.

The genetic landscape of chordoma harbors additional mutations. Sequencing analysis of 104 sporadic chordoma demonstrated PI3K signaling pathway mutations in 16% of cases as well as mutations in genes responsible for chromatin remodeling in 17%. These mutations represent plausible driver mutations for the tumor ^15^. Of unclear significance was the presence of mutations within the lysosomal trafficking regulator protein LYST in 10% of samples. Although this may represent a novel oncogene for chordoma formation, further investigation is warranted. On the other hand, in almost half of studied samples that were genetically sequenced, no plausible genetic drivers for mutation were identified ^15^. In light of these recent discoveries, extra-genetic abnormalities have been postulated ^15^, and while demonstrable progress has been made in the molecular understanding of these tumors, further evaluation including attention to epigenetic and other transcriptional regulatory mechanisms remains necessary. Molecular study may also have significant implications in the prognostication of tumor response to chemotherapy or radiation surgery ^16^.

## Treatment

Perhaps because of their indolent nature, chordomas are resistant to treatment with conventional cytotoxic chemotherapy regimens ^17^. The bedrock of their therapy remains surgical resection with a goal of total resection of the disease. Special care is given to excising the tumor en bloc when possible because of high rates of local recurrence after surgery, which appears to be due to cellular spilling if the tumor capsule is violated. This high recurrence rate, despite a misconception as a benign tumor, makes postoperative prognosis similar to that of malignant lesions ^18, 19^. Chordomas should be treated as locally malignant masses with a potential for metastasis. Published surgical series exploring patient outcomes have highlighted the importance of the extent of resection, and particularly gross total resection, as conferring a survival benefit ^20– 26^. As such, surgical advances have focused on approaches to these masses that facilitate maximal safe resection. For skull base chordomas in particular, the widespread use of the endoscopic endonasal approach has improved rates of gross total resection and decreased surgical morbidity compared to trans-cranial or trans-oral routes ^27, 28^. This holds true for spinal disease where techniques allowing en bloc resection and combined approaches allowing surgeons to obtain negative margins have been shown to improve disease-free as well as overall survival ^22, 24, 26, 29^. Unfortunately, despite technical breakthroughs and recent efforts to pursue aggressive surgical management, patient outcomes remain disappointing. This makes chordoma an ideal theoretical candidate for strategies that could potentially reduce the tumor burden preoperatively, or sterilize postoperative resection beds from tumoral cells, such as radiation therapy.

The use of adjuvant radiotherapy has increased over time, particularly with the availability and use of particulate therapy such as proton or carbon beams, over traditional photon therapy. The treatment of chordomas with radiotherapy was initially hampered by the large dose sizes required to achieve a biological response. These doses, in the range of 70 Gy, posed a significant risk of damaging surrounding critical neurologic structures including the spinal cord, brainstem, and optic pathways ^30, 31^. Charged particles, however, have the advantage of a more rapid radiation falloff beyond the target zone, allowing for larger doses to be delivered with less beam spill-out into the surrounding structures ^31, 32^. Despite a more favorable profile of particle-based therapies compared to photon therapies, reported complication rates can still be as high as 20% ^32^.

Carbon ion therapy also holds promise as another particle-based treatment option. Although clinical experience with it is still limited compared to proton beam therapy, the heavier mass of carbon atoms can theoretically confer a biological advantage with a more efficient energy transfer to the target tissue and even less contamination of the surrounding structures ^33^. Despite these theoretical benefits, clinical experience has yet to establish a definitive difference between the two modalities. Retrospective analysis suggests similar survival patterns and complication profiles between the two modalities ^34^. Further evaluation will be necessary as these therapies become increasingly available to determine whether the biologic advantages of carbon ion therapy translate to clinical outcomes.

Despite aggressive surgical treatment and adjuvant radiotherapy, recurrence rates remain upward of 50%, with many of these cases occurring late, sometimes more than 5 years after the initial intervention ^35– 38^, although some more recent stereotactic radiosurgery protocols managed to achieve a 5-year local recurrence-free survival upward of 80% ^39^. These timeframes make prolonged surveillance necessary, even in cases where gross total resection was achieved. In 2017, the Chordoma Global Consensus Group released a statement addressing the treatment of loco-regional recurrence ^35^. Hampered by the paucity of strong evidence regarding the management of recurrence, they suggest considering salvage surgery and radiation therapy in settings where performance status and morbidity are not prohibitive ^35^. While formal protocols for surveillance after surgery are not yet in place, it is reasonable to recommend annual magnetic resonance imaging with contrast administration for a period of 5 years ^29^. Data supporting a surveillance modality for longer follow-up periods is unclear.

## Future directions

Although surgery and stereotactic radiation have accumulated a solid record as standards of care, the promise of a definitive future solution resides in tailored targeted molecular and genetic compounds. Early data suggesting a role for these therapies exist, although larger-scale studies of efficacy are not yet underway ^40^. The Chordoma Foundation hosts a consistently updated list of active clinical trials on their website ^41^.

Brachyury has been targeted as a postulated driver of chordoma formation via vector-based vaccines. Preclinical and phase I trials of these vaccines have demonstrated the potential ability to promote Brachyury-specific T-cell activation with limited adverse events ^42^. Cellular signaling molecules including epithelial-derived growth factor receptors (EGFRs) and platelet-derived growth factor receptors (PDGFRs) are also potential targets. In the case of EGFR, *in vitro* studies using established chordoma cell lines as well as patient-derived xenografts demonstrated a biological efficacy with molecular inhibitors ^43, 44^. These findings led to a phase II study of the EFGR inhibitor lapatinib in patients with advanced disease. Although the effect was only modest, with a third of trial patients showing a partial response over the treatment course, the EGFR remains a potential therapeutic target ^45^. Similarly, PDGFR expression has been found among cohorts of chordoma patients, leading to a phase II trial of imatinib. The response was again modest, with 64% of patients demonstrating clinical benefit, which was defined as the achievement of a partial response or as disease stability for a duration of at least 6 months ^46^.

Immune checkpoint inhibitor molecules have also been a target of interest in the treatment of chordoma. These drugs have demonstrated remarkable success in the management of other malignancies ^47^. Programmed cell death ligand 1 (PD-L1) is one of the immune checkpoint targets and has an FDA-approved monoclonal antibody inhibitor, nivolumab. When bound to its ligand, PD-L1 triggers a cascade leading to decreased immune signaling and T-cell apoptosis, which modulates the immune response against malignant cells ^48^. Evaluation of chordoma samples has shown PD-L1 expression in over 90% of 74 samples evaluated in a tissue array ^49^. At present, the evidence of clinical benefit with nivolumab treatment is anecdotal but has prompted further investigation with three clinical trials currently open to evaluate its benefit ^49^.

While these findings are still preliminary, the presence of these molecular targets gives us hope for a biologic approach to the treatment of chordoma. As the horizons of genomic, epigenomic, and proteomic analysis progress, therapies based on these discoveries are likely to play an increased role in the management of this complex disease. The recommended approach to treat chordomas currently remains multidisciplinary, as collaborative efforts among surgery, radiation therapy, immunotherapy, and genetics, at medical centers that have experience managing the intricacies of the disease, are necessary to provide patients with the optimal chances of survival while minimizing treatment morbidity.
